# Analyses of methylomes of upland and lowland switchgrass (*Panicum virgatum*) ecotypes using MeDIP-seq and BS-seq

**DOI:** 10.1186/s12864-017-4218-0

**Published:** 2017-11-07

**Authors:** Mollee Dworkin, Shaojun Xie, Malay Saha, Jyothi Thimmapuram, Venu (Kal) Kalavacharla

**Affiliations:** 1 Molecular Genetics and Epigenomics Laboratory, Delaware State University, Dover, DE 19901 USA; 2 Bioinformatics Core, Purdue University, West Lafayette, IN 47907 USA; 3 Samuel Roberts Noble Foundation Inc., Ardmore, OK 73401 USA; 4 Center for Integrated Biological and Environmental Research (CIBER), Delaware State University, Dover, DE 19901 USA

**Keywords:** *Panicum virgatum*, Switchgrass, Methylome, Whole genome bisulfite-sequencing, MeDIP-Seq

## Abstract

**Background:**

Switchgrass is a crop with many desirable traits for bioenergy production. Plant genomes have high DNA methylation levels throughout genes and transposable elements and DNA methylation is known to play a role in silencing transposable elements. Here we analyzed methylomes in two switchgrass genotypes AP13 and VS16. AP13 is derived from a lowland ecotype and VS16, typically considered drought-tolerant, is derived from an upland ecotype, both genotypes are tetraploid (2n = 4× = 36).

**Results:**

Methylated DNA immunoprecipitation-sequencing (MeDIP-seq) and bisulfite-sequencing (BS-seq) were used to profile DNA methylation in genomic features of AP13 and VS16. The methylation patterns in genes and transposable elements were similar to other plants, however, overall CHH methylation levels were comparatively low. Differentially methylated regions (DMRs) were assessed and a total of 1777 CG-DMRs, 573 CHG-DMRs, and 3 CHH-DMRs were detected between the two genotypes. TEs and their flanking regions were higher than that of genic regions. Different types of TEs had different methylation patterns, but the two LTRs (Copia and Gypsy) were similarly methylated, while LINEs and DNA transposons typically had different methylation patterns. MeDIP-seq data was compared to BS-seq data and most of the peaks generated by MeDIP-seq were confirmed to be highly methylated by BS-seq.

**Conclusions:**

DNA methylation in switchgrass genotypes obtained from the two ecotypes were found similar. Collinear gene pairs in two subgenomes (A and B) were not significantly differentially methylated. Both BS-seq and MeDIP-seq methodologies were found effective. Methylation levels were highest at CG and least in CHH. Increased DNA methylation was seen in TEs compared to genic regions. Exploitation of TE methylations can be a viable option in future crop improvement.

**Electronic supplementary material:**

The online version of this article (10.1186/s12864-017-4218-0) contains supplementary material, which is available to authorized users.

## Background

As the demand for food and energy increases, the need for renewable energy sources based on non-food crops is becoming a necessity. Switchgrass is viewed as an important crop in terms of biofuel-producing potential, with minimal input [1, 2]. Work on bioenergy crops in the late 1980s and 1990s revealed that switchgrass was among the crops with the highest potential as a dedicated next-generation feedstock [1]. Switchgrass has the capacity to grow on marginal lands, which are not used for food crops.

Switchgrass genotypes are categorized as either upland or lowland ecotypes. Members of the upland ecotype are usually tetraploid or octoploid, have more narrow stems, and are better adapted to drought conditions in the northern United States [3, 4]. The lowland ecotype is usually tetraploid, with taller and wider stems, and are better suited for wet conditions with periodic flooding in southern parts of the U.S. [3, 4]. Improvement of both ecotypes is important and necessary for use as a bioenergy feedstock.

DNA methylation is one of the primary mechanisms affecting DNA structure and organization [5]. DNA methylation plays a significant role in the regulation of gene expression, including limiting expression of transposons [6, 7]. While highly expressed genes can contain relatively high levels of DNA methylation within the gene body, methylation at transcription start sites (TSSs) or promoters are typically linked to decreased gene expression [8, 9].

Plants have three contexts that are frequently methylated throughout their genomes, CG, CHG, and CHH [9, 10]. Recent research shows specific functions of non-CG DNA methylation in mammals; however, the extent is typically far less than what is found in plants and is highly tissue-specific [11]. Plant-specific enzymes RNA Polymerase IV and V are responsible for the introduction of new DNA methylation via siRNAs and lncRNAs [10].

The effects of DNA methylation on gene expression and chromosomal organization in plants and other eukaryotes are well-known [5, 10]. Transposable elements (TEs) are ubiquitous throughout the genome and usually under tight control by DNA methylation for silencing [12]. There are several different methods that are used for profiling genome-wide or targeted DNA methylation. In this study, we examine two different methylome sequencing technologies; methylated DNA immunoprecipitation-sequencing (MeDIP-seq), which employs an anti-cytosine antibody, and bisulfite-sequencing (BS-seq), a single-base resolution technique, on AP13 and VS16 genotypes of switchgrass. Our goal is to determine differentially methylated regions (DMRs), to distinguish methylation patterns in TEs, and to compare the BS-seq and MeDIP-seq methodologies.

## Results

### Data collection and preprocessing

Deep sequencing of two bisulfite-seq libraries in AP13 and VS16 switchgrass resulted in a total of 707,684,647-50 bp paired-end Illumina reads (Table 1). MeDIP-seq: A total of 462,293,537-50 bp single-end reads were generated from an input library and three MeDIP-seq libraries for each genotype, (4 + 4 = eight separate libraries). The raw reads were trimmed, filtered, and high quality reads collected were aligned to the unpublished reference genome *Panicum virgatum* V2.1 accessed January 2016 (Schmutz et al., unpublished). Of these, only uniquely mapped reads with ≤2 mis-matches were further used in analysis.

**Table 1:** Reads and Mapping Summary of BS-Seq and MeDIP-Seq Data

Sample	Total reads	Mapping rate
VS16 BS-Seq	346,153,526 pairs	41%
AP13 BS-Seq	361,531,121 pairs	45%
VS16 MeDIP Input	32,697,930	75%
VS16 MeDIP_1	52,396,117	72%
VS16 MeDIP_2	72,502,774	63%
VS16 MeDIP_3	62,601,002	63%
AP13 MeDIP Input	51,348,721	71%
AP13 MeDIP_1	69,288,317	66%
AP13 MeDIP_2	64,084,865	74%
AP13 MeDIP_3	57,373,811	79%
VS16 MeDIP Combined	187,499,893	66%
AP13 MeDIP Combined	190,746,993	73%

### Genome-wide DNA methylation patterns

**Fig. 1:**
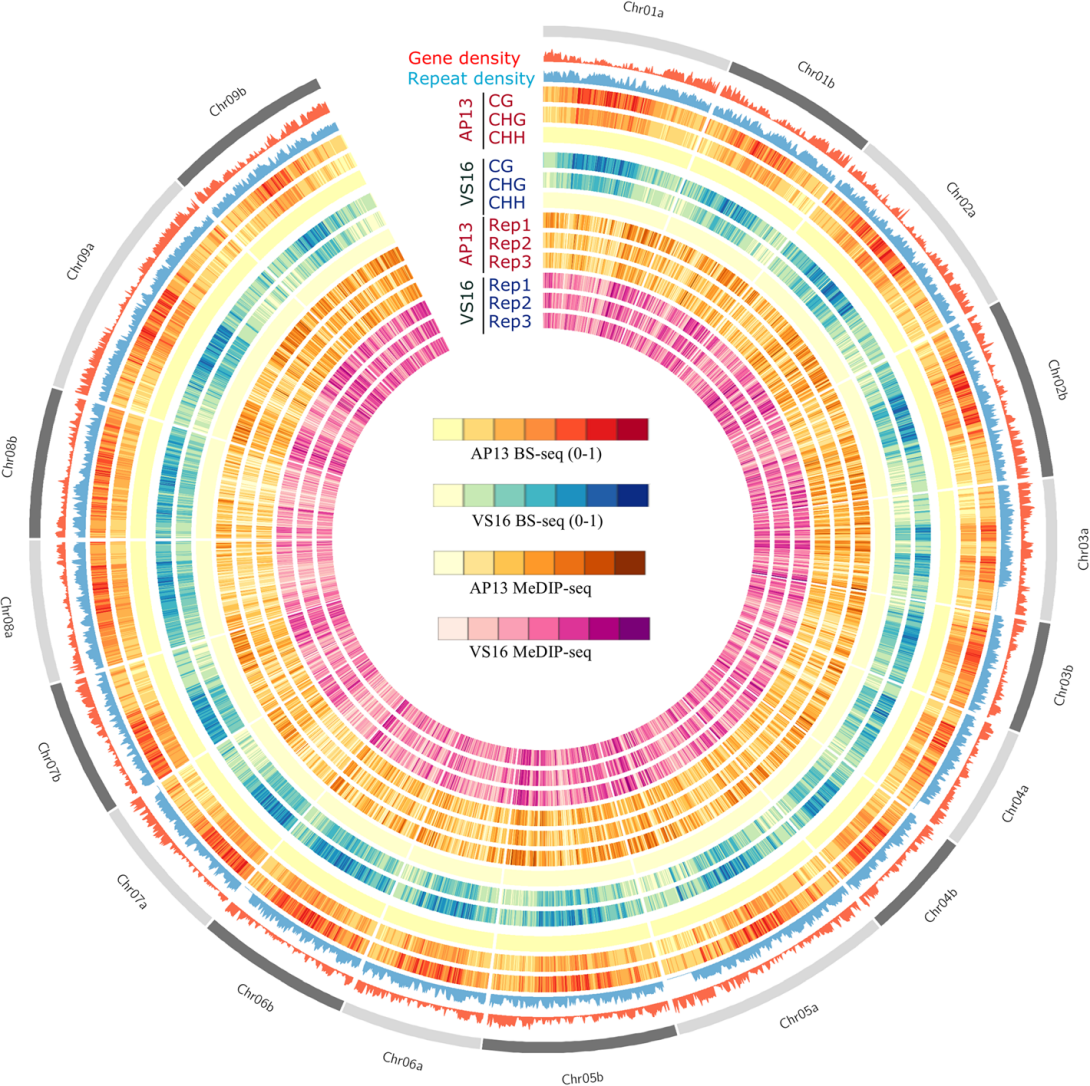
Circos plot of gene density, TE density, and methylation levels of CG, CHG and CHH context across each chromosome of switchgrass genome. Each MeDIP-seq replicate and BS-seq sample, by context, are displayed. The color key for MeDIP-seq rings indicates the density of peak numbers in each sample, not the methylation levels. The methylation signals detected by MeDIP are combinations of CG, CHG and CHH methylation. In BS-Seq each context is represented separately

**Fig. 2:**
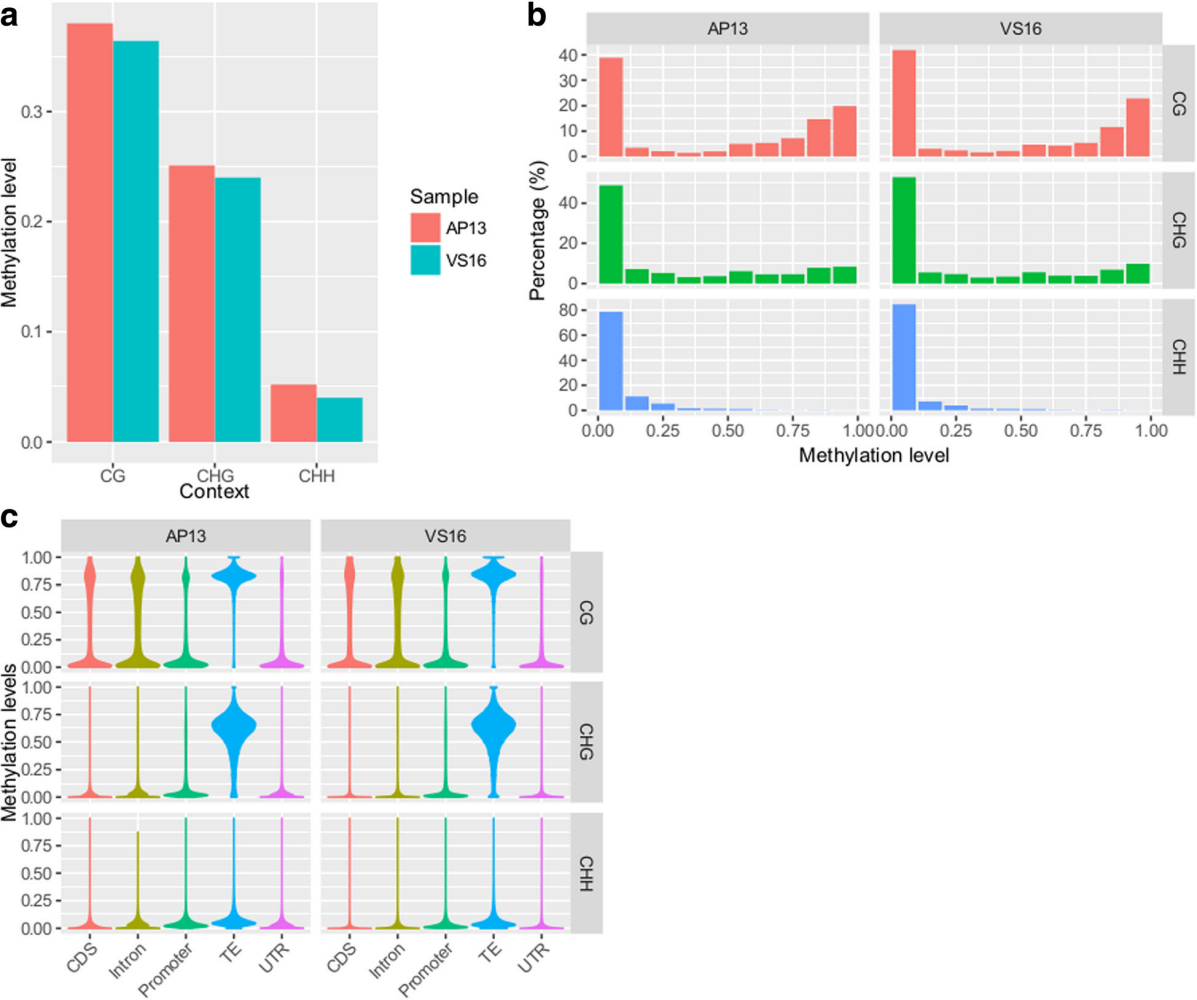
Global methylation levels, distribution, and across genomic features for CG, CHG and CHH contexts. **a** Methylation levels are shown for AP13 and VS16, derived from BS-seq data. **b** Percentages are shown for AP13 and VS16, derived from BS-seq data. **c** Violin plot of methylation level in different features

Methylation profiles for each chromosome are shown in heat maps separated by genotype, methylation pattern (for bisulfite-seq), and MeDIP-seq replicate (Fig. 1). Repeat-rich regions were more highly methylated than gene-rich regions. The CG context was the most methylated sequence, followed by CHG, and CHH, as determined by whole genome bisulfite-sequencing (BS-seq). Genome-wide weighted DNA methylation levels were calculated (0.0 indicating no methylation and 1.0 indicating full methylation), and revealed 0.38 and 0.36 methylation in the CG context for AP13 and VS16, respectively. CHG methylation was 0.25 and 0.24 and CHH methylation was 0.05 and 0.04, for AP13 and VS16, respectively (Fig. 2a).

Global methylation level distributions showed that around 40% of CG sites had low methylation levels (<0.2) and 48% showed high methylation levels (>0.6). About 51% of CHG sites had low and 25% had high methylation levels. CHH site levels were overall very low, with about 82% in the low methylation level category and only 1% had high methylation levels (Fig. 2b).

Methylation levels for genomic features were analyzed by dividing the genome into the following categories: transposable elements (TE), promoter, coding sequence (CDS), untranslated region (UTR), and introns. TEs were found to be the most methylated in the CG context, followed by introns, CDS, promoters, and UTRs. Only TEs showed high CHG methylation levels and CHH levels were low across all regions (Fig. 2c).

### DNA methylation patterns across genic and TE regions

**Fig. 3:**
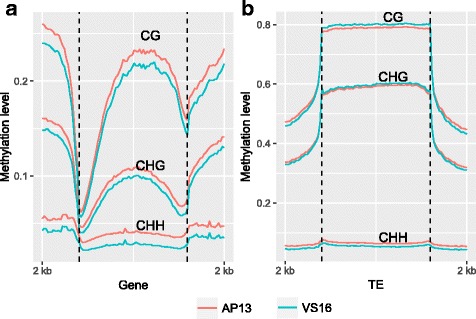
Meta-plots of DNA methylation level across genes and TEs. AP13 and VS16 methylation levels are shown for 2 kb upstream and downstream regions, as well as **a**) in the gene body and **b**) in the TE body

**Fig. 4:**
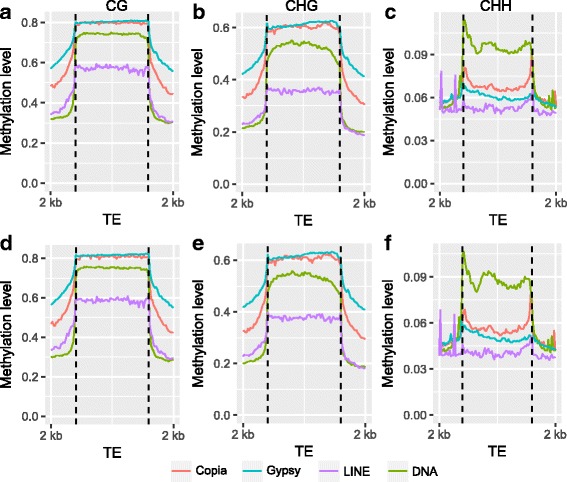
Methylation patterns of different types of TEs for AP13 (**a**-**c**) and VS16 (**d**-**f**). **a** and **d** show CG methylation levels, **b** and **e** show CHG methylation levels, and **c** and **f** show CHH methylation levels in Copia, Gypsy, LINE, and DNA TEs

DNA methylation in the upstream and downstream regions of genes in all three contexts were either higher or about the same as the highest genic methylation levels, while a sharp drop was observed at the TSS and TTS (Fig. 3). When the methylation levels of genes without intronic TEs were profiled, overall gene body methylation was seen to decrease, especially in the CHG context (Additional file 1: Figure S1). Meta-plots of DNA methylation levels across genes and TEs show there were comparatively higher CG and CHG methylation levels upstream and downstream of TEs than genes, TEs themselves were more highly methylated than upstream and downstream regions (Fig. 3). Methylation levels across different types of TEs were examined for: LTR-Copia, LTR-Gypsy, LINEs, and DNA transposons. Methylation levels in the four classes of TEs are shown for CG, CHG, and CHH in AP13 (Fig. 4a-c), and VS16 (Fig. 4d-f). The two LTR types (Copia and Gypsy) had similar methylation patterns in all three contexts within TE bodies. However, in the 2 kb upstream and downstream regions, Gypsy CG and CHG methylation levels were higher than Copia (Fig. 4). Overall CG and CHG methylation levels were lowest in LINEs, higher in DNA transposons, and highest in the LTRs. DNA transposons showed highest CHH methylation in the body regions (Fig. 4c and f), although CHH methylation levels were low.

### Comparison of AP13 and VS16 DNA methylation and identification of differentially methylated regions (DMRs)

Differentially methylated cytosines (DMCs) were detected for each context between AP13 and VS16. There were 116,600 CGs, 37,243 CHGs, and 840 CHHs identified to be differentially methylated. Since DNA methylation typically does not occur randomly, DMCs that were in close proximity (within 100 bp) were merged into differentially methylated regions (DMRs). Hypermethylation indicates higher methylation in VS16 and hypomethylation indicates higher methylation in AP13. A total of 1777 CG-DMRs (876 hyper- and 901 hypo-methylations), 573 CHG-DMRs (439 hyper- and 134 hypo-methylations), and 3 hypo CHH-DMRs were identified (Table 2).

**Table 2:** Derivation of differentially methylated cytosines (DMCs) and differentially methylated regions (DMRs) from BS-Seq Data

	CG	CHG	CHH
Cytosine sites with depth > =5	43,645,671	40,463,671	116,652,010
Cytosine sites with *P* < 0.05	2,476,686	2,058,714	1,611,070
DMC (Adjusted P < 0.05)	116,600	37,243	840
DMC cluster (> = 4 DMC & > =50 bps)	2832	615	4
DMR	1777	573	3

**Fig. 5:**
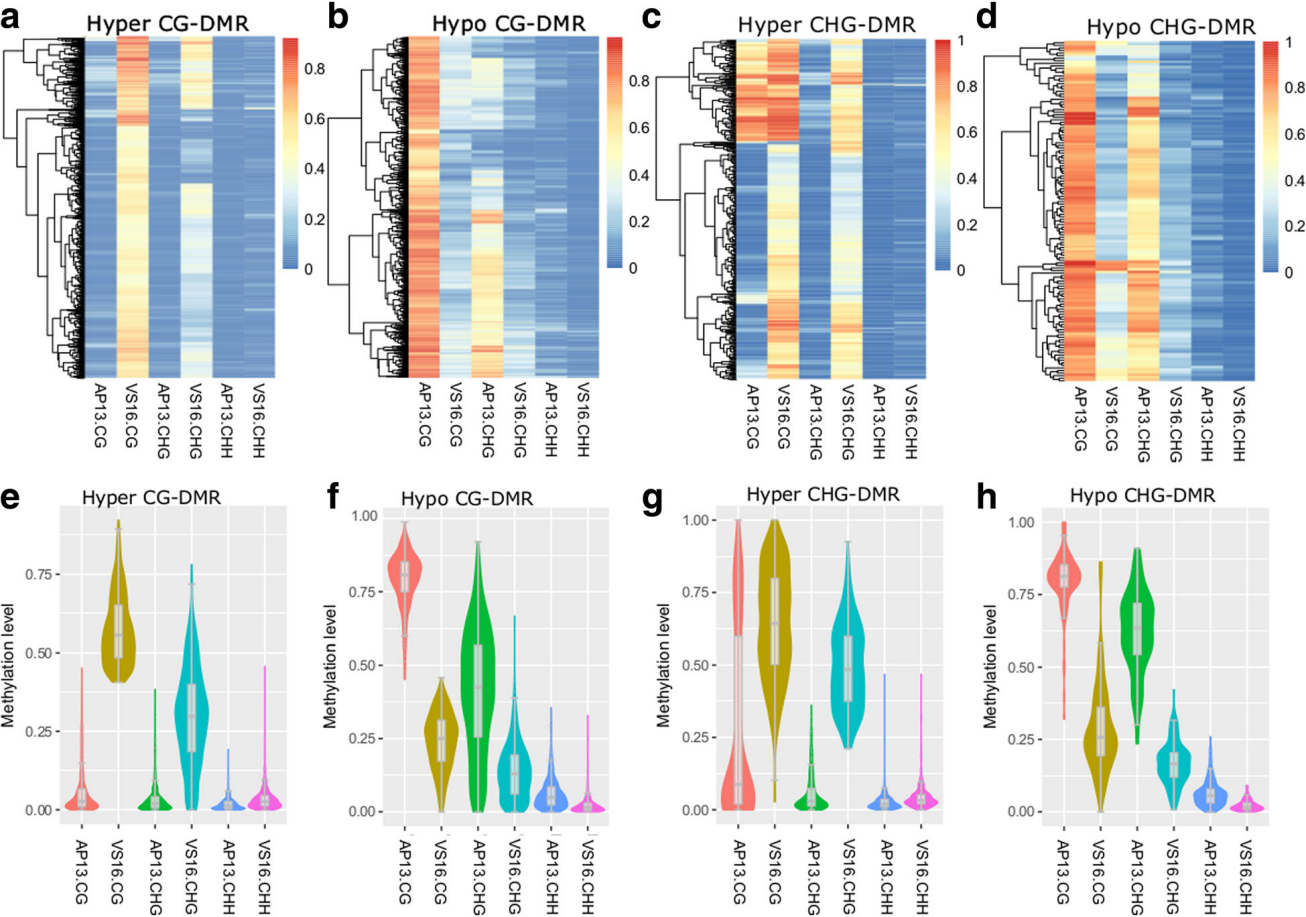
Heat maps (**a**-**d**) and Violin-boxplots (**e**-**h**) of DNA methylation levels of CG- and CHG-DMRs

We further found that hypermethylated CG-DMRs coincided with hypermethylation at CHG and CHH regions (Fig. 5). Hypermethylated CHG-DMRs also showed higher CG methylation in VS16 (Fig. 5c and g). Similar patterns were seen in hypomethylated CG-DMRs (Fig. 5b and f) and CHG-DMRs (Fig. 5d and h). The association between CHH and CG- and CHG-DMRs is not strong, but is still visible.

**Fig. 6:**
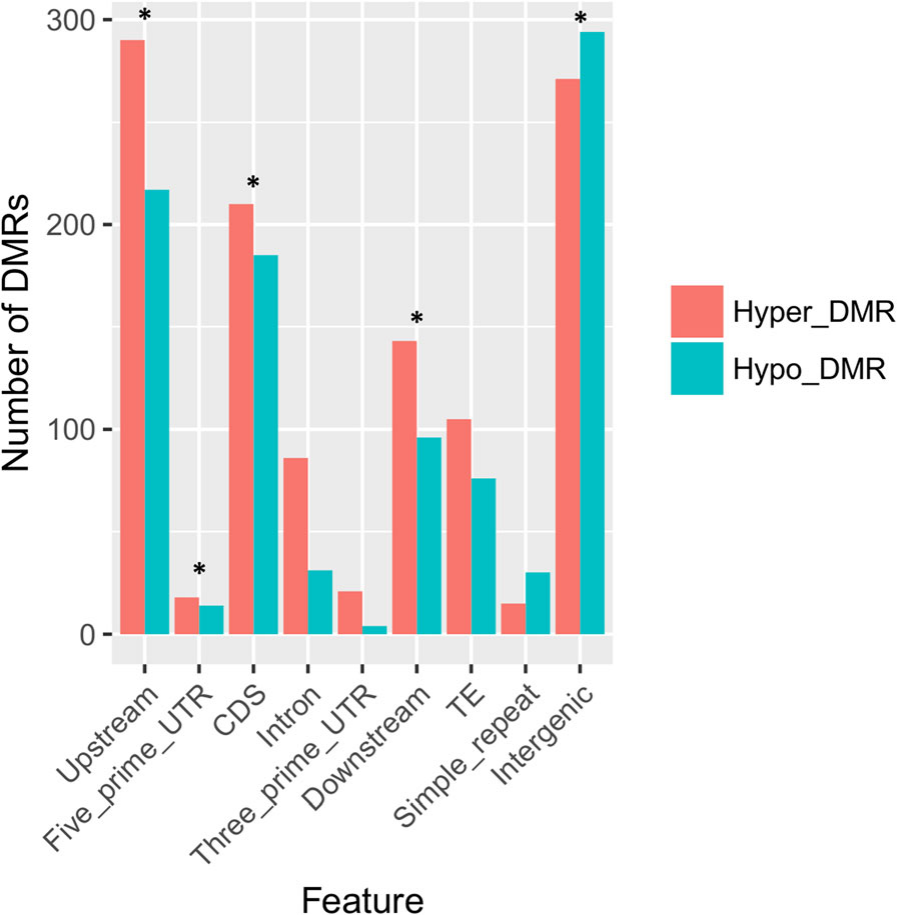
Distribution of DMRs based on genomic features. Asterisks indicate genomic features with overrepresentation in DMRs

**Fig. 7:**
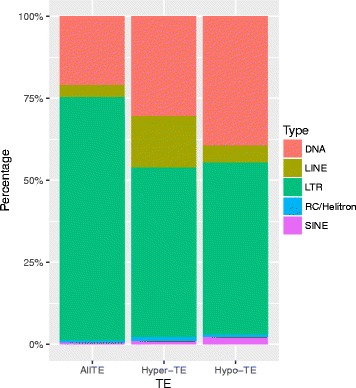
Distribution of types of TEs that were associated with DMRs

Since DMRs were typically seen to have differences in all three contexts, DMRs were merged for downstream analysis. A total of 1159 hypermethylated DMRs and 947 hypomethylated DMRs were found. DMRs were overrepresented in CDS, upstream and downstream regions of genes, 5′ UTR and intergenic regions (Fig. 6, Additional file 2: Table S1; hypergeometric test). TEs that were found closest to DMRs were LTRs, DNA transposons, LINEs, SINEs, and RC/Helitron (Fig. 7). The average length of these TEs is shorter than that of all TEs across the genome (Additional file 3: Figure S2).

Genes located within 2 kb of DMRs were extracted. A total of 805 genes were extracted from near hypermethylated-DMRs and 624 genes were extracted from near hypomethylated-DMRs. Analysis of gene ontology (GO) terms did not yield enrichment among these genes (Additional file 4: Table S2). The genes within 2 kb of hyper-DMRs were related to various biological processes, including cellular amino acid and derivative metabolic process, cellular amino acid metabolic process, DNA repair and response to DNA damage stimulus, etc. Also they had various molecular functions, including transferase activity, transferring alkyl or aryl (other than methyl) groups, nucleotidyltransferase activity, RNA polymerase activity, nucleotide binding, etc. For cellular component, these genes were involved in ribonucleoprotein complex, intracellular nonmembrane-bounded organelle, intracellular part, etc. The genes with 2 kb of hypo-DMRs were involved in biological processes including RNA biosynthetic process, regulation of metabolic process, phosphorylation, and regulation of cellular metabolic process, etc. For molecular functions, these genes were involved in nucleotide binding transferase activity, transferase activity, transferring one-carbon groups, transferase activity, transferring acyl groups, etc. For cellular component, these genes were involved in integral to membrane, membrane, intracellular membrane-bounded organelle, intracellular part, etc. (Additional file 5: Table S3).

AP13 and VS16 are tetraploid with 2n = 4× = 36 chromosomes. In order to compare the A and B subgenomes, collinear gene pairs were identified. A total of 27,144 pairs were found. There was no statistical difference in DNA methylation levels found between subgenomes A and (Additional file 6: Figure S3).

### Methylated DNA Immunoprecipitation-sequencing (MeDIP-Seq)

**Fig. 8:**
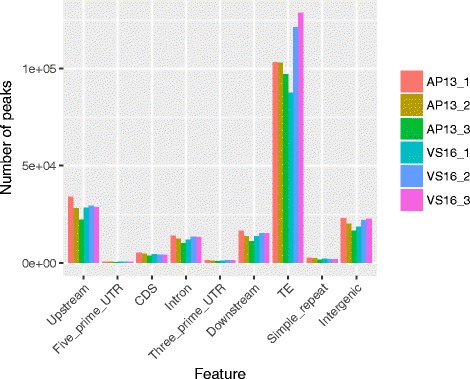
Distribution of peaks based on genomic features

MeDIP-seq was performed in triplicate on leaves from three clonal ramets, for both VS16 and AP13. In AP13, there were 200,791 peaks in replicate one, 186,152 peaks in replicate two, and 163,754 peaks in replicate three were identified. In VS16, there were 168,623 peaks in replicate one, 209,611 peaks in replicate two, and 216,755 peaks in replicate three were identified. These peaks were enriched in TEs (Fig. 8). There were 18,289 common peaks between AP13 and VS16. AP13 had 1219 regions with significantly higher methylation, while VS16 was found to have 63 regions with higher methylation. The remaining peaks were not statistically enriched in either genotype. Total MeDIP-seq peaks in localized regions from combining replicates in each genotype are summarized in Table 3. Peaks per Mb were determined in total peaks found in both genotypes, showing generally which chromosomes are more or less methylated than average. Based on this method, Chr09b is the highest methylated chromosome, followed by Chr05b and Chr08b has the least peaks per Mb of DNA, followed by Chr08a (Table 3).

**Table 3:** Peaks obtained from MeDIP-Seq data combined across replicates and their distribution in switchgrass genome

	AP13		VS16		Chr length (Mb)	AP13		VS16	
Chr	Total peaks	Annotated	Total peaks	Annotated		Total peaks/Mb	Annotated peaks/Mb	Total peaks/Mb	Annotated peaks/Mb
1a	9383	1298	6610	953	97.81	95.93	13.27	67.58	9.74
1b	8176	1230	5673	907	80.38	101.71	15.30	70.57	11.28
2a	10,727	1502	7608	1165	103.95	103.20	14.45	73.19	11.21
2b	9030	1296	6631	1035	93.55	96.52	13.85	70.88	11.06
3a	7695	1339	5610	1004	73.00	105.41	18.34	76.85	13.75
3b	5355	729	3697	533	57.82	92.62	12.61	63.94	9.22
4a	6214	949	4312	686	63.56	97.76	14.93	67.84	10.79
4b	5599	820	3813	584	56.30	99.44	14.56	67.72	10.37
5a	11,457	1836	8246	1406	116.48	98.36	15.76	70.79	12.07
5b	10,597	1575	7576	1200	100.40	105.55	15.69	75.46	11.95
6a	6429	957	4663	744	72.90	88.19	13.13	63.97	10.21
6b	7013	801	5118	626	80.13	87.53	10.00	63.87	7.81
7a	6179	953	4392	731	75.76	81.56	12.58	57.97	9.65
7b	6646	978	4721	738	70.77	93.91	13.82	66.71	10.40
8a	5524	720	3774	520	73.87	74.78	9.75	51.09	7.04
8b	5968	733	3762	517	77.82	76.69	9.42	48.34	6.64
9a	12,929	1845	9148	1397	122.78	105.30	15.03	74.51	11.38
9b	9541	1460	7322	1189	87.97	108.46	16.60	83.23	13.52

Overall, more peaks were found in AP13 (144,462 excluding scaffold) than in VS16 (102,676) when replicate samples were combined (Table 3). Despite the difference in the number of peaks, the peak density across chromosomes was similar between VS16 and AP13. Approximately 14% of the total MeDIP-seq peaks were located within annotated regions (promoter or genic) in both genotypes (Table 3).

### Comparison of BS-Seq and MeDIP-Seq

Peaks obtained from MeDIP-seq and their corresponding single-base methylation sites derived from BS-seq, were compared. This allowed us to correlate the peaks identified in likely methylated regions and to compare the two methylome sequencing work flows. In order to compare the techniques, there were three assumptions: a) The regions of common peaks derived from MeDIP-seq analysis also exhibit high methylation in BS-seq in both AP13 and VS16, b) The 1219 regions that showed higher DNA methylation in AP13 based on MeDIP-seq results should have higher DNA methylation levels in AP13 than VS16 in BS-seq data, and c) The 63 regions that showed higher DNA methylation in VS16 based on MeDIP-seq results should have higher DNA methylation levels in VS16 than AP13 in BS-seq data.

**Fig. 9:**
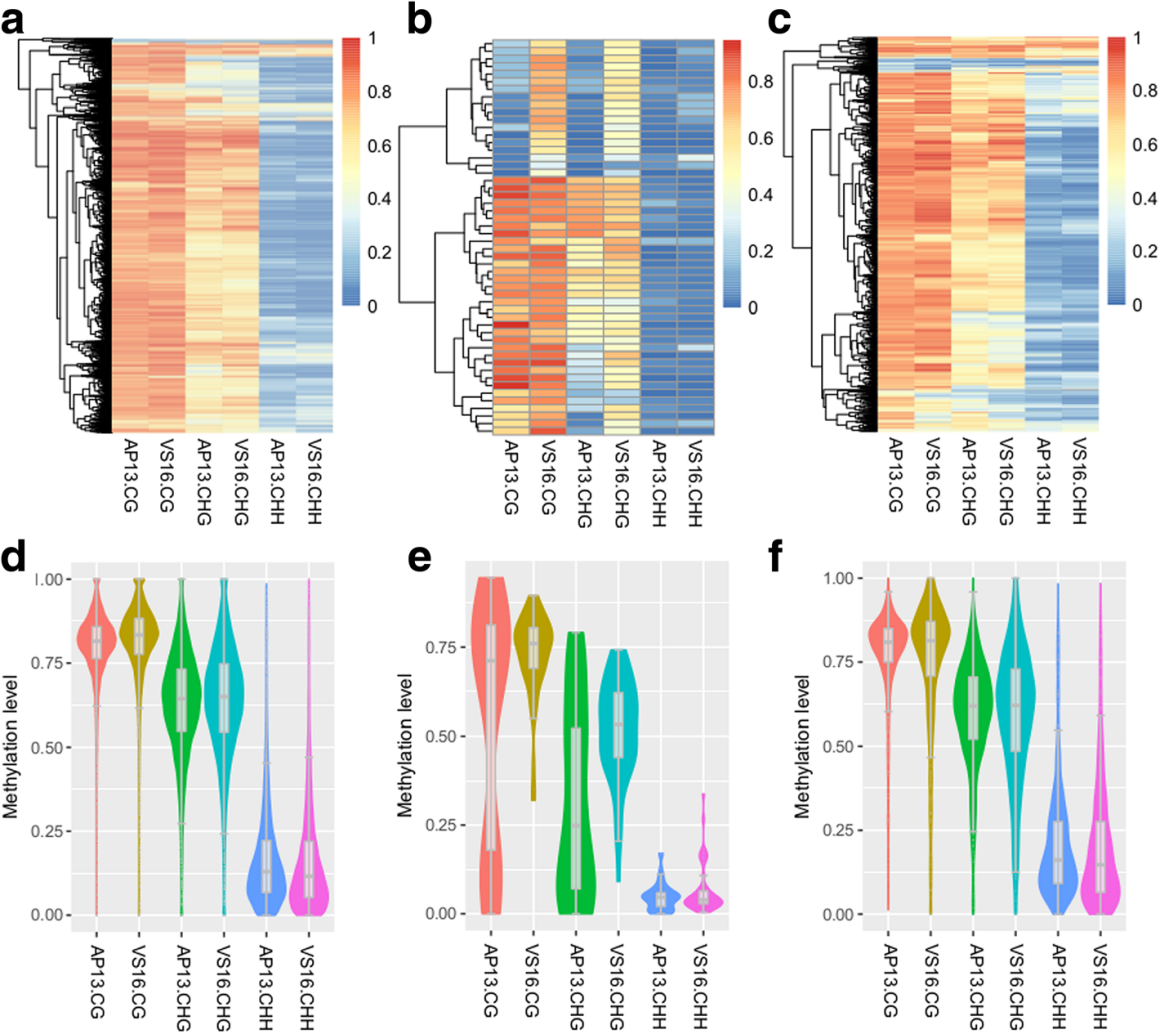
Heat map and Violin-boxplots representing DNA methylation levels for common (**a** and **d**), hypermethylated peaks and differential peaks (**b**, **c**, **e** and **f**) in CG, CHG and CHH contexts

To confirm this, CG, CHG, and CHH methylation levels were calculated for the common and differentially methylated peaks based on the BS-seq data. Heat maps confirmed that the common regions showed similar patterns in each context in both AP13 and VS16 (Fig. 9a and d). The vast majority of CG and CHG site methylation levels of the common peaks derived from MeDIP-seq had methylation levels over 0.50, as determined by BS-seq. This indicated the regions detected by MeDIP-seq were much more methylated than global DNA methylation levels (Additional file 7: Figure S4) compared to Fig. 2b. The 63 regions that were more highly methylated in VS16 than AP13 based on MeDIP-seq, were also found to be significantly more methylated in BS-seq (T-test; P: 0.00016 for CG, 8.567e-06 for CHG and 0.035 for CHH), although for some regions VS16 did not exhibit higher DNA methylation levels (Fig. 9b and e). In the 1219 regions showing higher DNA methylation in AP13, obvious higher methylation was not seen in all three contexts (Fig. 9c and f). However, the CG context had significantly higher methylation levels in AP13 than in VS16 (*P*-value: 0.018).

**Fig. 10:**
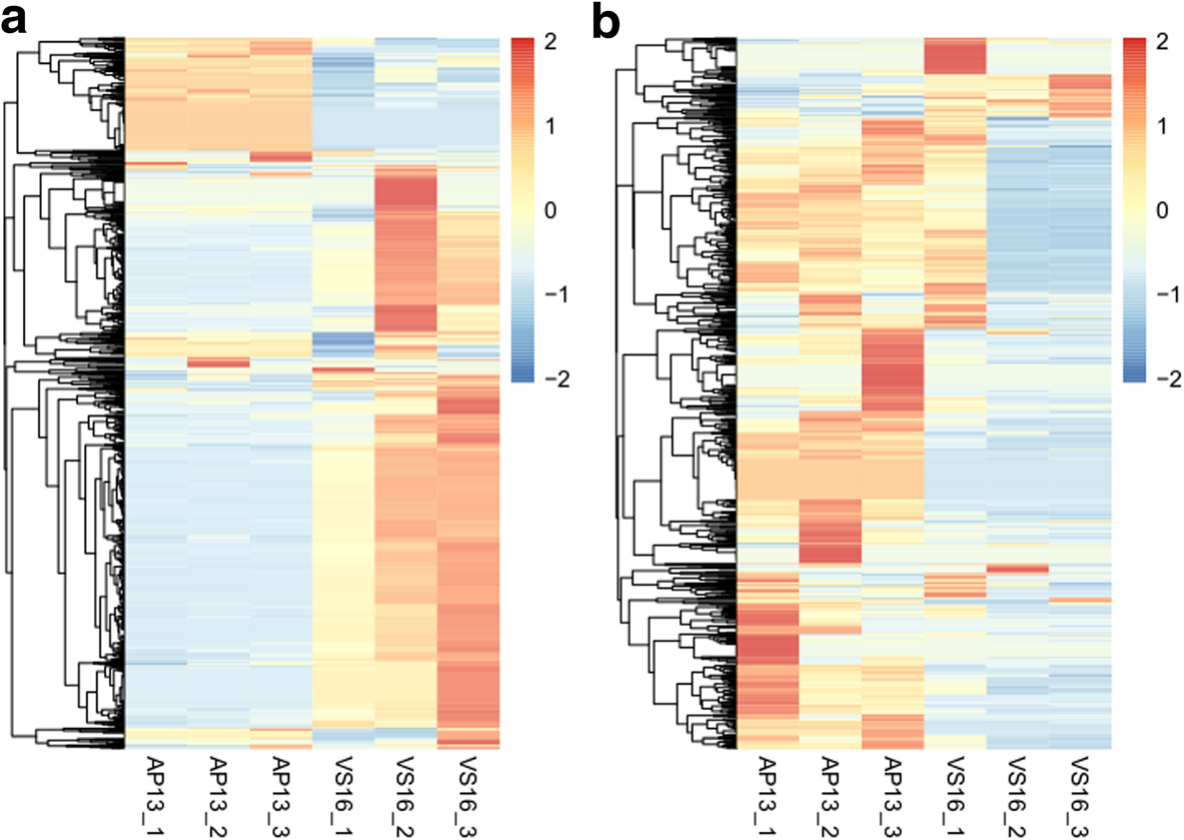
Heat map of MeDIP-Seq signals for hypermethylated-DMRs (**a**) and hypomethylated-DMRs (**b**). The color key for indicates the density of peak numbers in each sample, not the methylation levels

The DMRs identified from BS-seq data were compared back to MeDIP-seq with two possible assumptions: a) Hypermethylated-DMRs should have higher MeDIP-seq peak signals in VS16 than in AP13 and b) Hypomethylated-DMRs should have higher MeDIP-seq peak signals in AP13 than in VS16. In fact this is the case and we found that MeDIP-seq signals in VS16 were higher than in AP13 for hyper-DMRs and hypo-DMRs showed lower MeDIP-seq signals in VS16 than in AP13 (Fig. 10). The overall differences in MeDIP-seq signals in the two genotypes were not large. Examples of a hypermethylated-DMR and hypomethylated-DMR with corresponding MeDIP-seq signals are shown (Additional file 8: Figure S5).

## Discussion

### Genome-wide DNA methylation patterns

DNA methylation was highest in the CG context, followed by CHG, and CHH. This pattern is similar to previously studied monocot and dicot species [9, 13]. However, while the methylation levels in this study differ from the recently published switchgrass Kanlow genotype [9], there are some reasons that may account for this; including the switchgrass genome version that was used for mapping (V2 in our study and V1 in the previous study), the reference genome resulted from sequencing AP13, and/or the effect of clonal propagation. Niederhuth et al. 2016 indicated a pattern in clonally propagated species showed lower methylation levels in the CHH genotype, perhaps it can affect global methylation levels. Gene and TE density are correlated with DNA methylation levels (Fig. 1). In areas where TE density is high and gene density is low, there are high levels of DNA methylation, as detected by BS-seq and MeDIP-seq. Overall methylation levels between AP13 and VS16 were similar in each context. VS16 methylation levels were consistently slightly lower than that of AP13 (Fig. 2a). This could be partially attributed to the fact that the reference genome used in this study was derived from AP13. There may be some genotype-specific SNPs or indels, which could affect alignment and proper DNA methylation level analyses [14]. In addition, the two genotypes represent the two switchgrass ecotypes, which has distinct geographical niches and DNA polymorphisms throughout the genomes [15]. Spontaneous deamination of methylated cytosines ultimately causes a thymine substitution, which would be considered an unmethylated CG site [14, 16]. The majority of spontaneous mutations found in an *Arabidopsis* study were C:G → A:T transitions [16].

Most CGs had either low (<0.2) or high (>0.6) methylation levels, and only 12% were methylated between these ranges (Fig. 2b). CHG and CHH methylation is more variable, with about half of CHGs having low methylation levels and 25% of CHG sites exhibiting high methylation. Methylation levels in different genomic features were very different. TEs were highly methylated relative to the other features examined (Fig. 2c). High TE methylation was expected, as epigenetic silencing is targeted to TEs to prevent their activity [11, 17]. CG methylation was highest in TEs, followed by intron, CDS, promoter, and UTR-a pattern consistent with many other plant species [9, 18].

### DNA methylation patterns across genic and TE regions

In all three contexts, a relatively higher methylation level was seen 2 kb upstream of the transcription start site (TSS) (Fig. 3a). A very sharp dip in methylation levels just prior to and at the TSS was seen. Methylation levels were seen to increase in the gene body with another dip in methylation levels towards the TTS. This TSS and TTS pattern is a common DNA methylation pattern found in *Arabidopsis* [13, 19], soybean [20], cassava [21], and maize [22] and many other plant species [9]. The relatively high gene body CHG methylation is likely due to pseudogenes and intronic TEs (Additional file 1: Figure S1).

The higher methylation levels of TE than that of genes is consistent with the generally repressive function of DNA methylation (Fig. 3b). The relatively higher methylation of regions flanking TEs than around genic regions suggests that DNA methylation has spread outside of TE bodies. It is well known that genome size is directly related to TE content [23]. Transposable elements play a key role in shaping genomes and largely contribute to major evolutionary changes [12]. Classes of TEs behave differently throughout the genome and the methylation levels of two long terminal repeats (LTRs) were analyzed. Methylation levels of retrotransposons and DNA transposons were examined. The two LTR types, Copia and Gypsy, were similarly methylated in TE bodies, but Gypsy had higher methylation levels in the upstream and downstream regions in both AP13 (Fig. 4a-c) and VS16 (Fig. 4d-f). Similar results were also observed in cassava [21]. These results indicate that DNA methylation of LTR transposons is very important for repression of TE activity and for genome integrity. Lower levels of DNA methylation of *GYPSY* retro-transposons could lead higher transposition activity and result in genome expansion [24]. Taken together, these results of gene and TEs are in general consistent with that of other plant species [13, 19, 20, 22, 25].

### Comparison of AP13 and VS16 DNA methylation

CG and CHG sites were highly overrepresented in the differentially methylated cytosine (DMC) analysis. This may be attributed to the higher statistical power in detecting CG and CHG sites, due to the typically higher methylation levels of CG and CHG sites compared to CHH sites [26]. DMCs were merged into regions (DMRs), since DNA methylation is known to occur non-randomly and is often found in clusters [27]. Hypermethylated-DMRs refer to differentially methylated regions that had higher methylation in VS16 than in AP13, and hypomethylated-DMRs had higher methylation in AP13 than in VS16. There were 1777 CG-DMRs (876 hypermethylated and 901 hypomethylated), 573 CHG-DMRs (439 hypermethylated and 134 hypomethylated), and 3 CHH-DMRs (all hypomethylated). It is likely that deeper bisulfite sequencing coverage may allow more DMCs in the CHH context to be observed among switchgrass genotypes (Fig. 5) [26]. The genotype AP13 was derived from a lowland switchgrass cultivar “Alamo” and VS16 was derived from the upland cultivar “Summer.” These genotypes have been maintained in a Noble Foundation greenhouse for the last 11 years through clonal ramets. Clonal propagation is very common in switchgrass and other plant species. A recent study shows that plants with a history of clonal propagation have comparatively lower CHH methylation levels, which may partially account for the low number of CHH- DMRs [9].

The hypermethylated and hypomethylated DMRs in each context were merged to form 1159 hypermethylated-DMRs and 947 hypomethylated-DMRs (Fig. 6). These regions were overrepresented in promoters, CDS, downstream regions of genes, TEs, and intergenic regions. Promoter methylation in plants is typically negatively correlated with lower gene expression. Very low and high levels of methylation in the gene body are also correlated with lower gene expression [28]. Gene bodies may also contain relatively higher DNA methylation levels due to regulation of alternative and cryptic promoters in plants and animals [28–30].

The genes located within 2 kb of DMRs (805 hypermethylated-DMRs and 624 hypomethylated-DMRs) were extracted and analyzed for enrichment of gene ontology (GO) terms. No terms were enriched in these genes, suggesting that DNA methylation is broadly regulating genes, as opposed to specific sets of genes.

### Methylated DNA Immunoprecipitation-sequencing (MeDIP-Seq)

Consistent with BS-seq data, MeDIP-seq peaks were enriched in TEs (Fig. 8).

The unlocalized portion of the genome (scaffold) has the highest average peak density (MeDIP-seq peaks/Mb of DNA). This may be due to the presence of sequences that are difficult to localize and/or highly repetitive, which are likely to have relatively higher levels of DNA methylation throughout. There is the possibility of four copies of each sequence, since there is an A and B genome, each with two sets in the nucleus [31].

Peaks per chromosome, peak density, and peaks in annotated were determined in VS16 and AP13 (Table 3). Overall, more peaks were found in AP13 than in VS16 when replicate samples were combined This difference could be due to the reference sequence being derived from AP13. Despite the difference in the number of peaks, the peak density across chromosomes was similar between VS16 and AP13.

### Comparison of BS-Seq and MeDIP-Seq

The vast majority of methylation levels for CG and CHG sites in areas that were detected by MeDIP-seq were higher than global DNA methylation levels (Additional file 7: Figure S4) compared to Fig. 3. There were 18,289 common peaks between AP13 and VS16 detected by MeDIP-seq, which were compared to the BS-seq data to confirm whether methylation levels were high in both genotypes (Fig. 9a and d). This confirms that MeDIP-seq detects broadly methylated regions in switchgrass. The 1219 enriched MeDIP-seq peaks in AP13 had only significantly higher methylation levels in the CG context, though the pattern was not visibly apparent (Fig. 9c and f). The 63 enriched MeDIP-seq peaks that were also found to be significantly more methylated in all three contexts and some regions were not found to have higher DNA methylation levels in VS16 (Fig. 9b and e) .

The DMRs from BS-seq were compared to peak enrichment in the MeDIP-seq data. Hypermethylated-DMRs were regions that were more highly methylated in VS16 than AP13, while hypomethylated-DMRs were more highly methylated in AP13 than VS16. This was mostly in agreement with MeDIP-seq signals as well (Fig. 10). However, in the 1159 hyper-DMRs, only 11 of them were also identified as VS16 upregulated peaks in MeDIP-seq data. In the 947 hypo-DMRs, only 8 of them were identified as AP13 upregulated peaks. Together, the result indicates that MeDIP-Seq detected methylated regions well, but did not perform as well, in detecting differential methylation between AP13 and VS16.

### Future application of TEs in plant breeding

Epigenetic information is considered as one possible source of missing heritability [32]. Epigenetic variations caused by TE insertions can lead to phenotypic variations, such as natural variation in floral symmetry [33], sex determination in melon by a transposon induced epigenetic variation [34], and abortion of male sexual organs because of DNA methylation variations caused by insertion of the hAT transposon upstream of the CmWIP1 gene [34]. In *Arabidopsis*, the *ONSEN* insertion yielded an abscisic acid-insensitive phenotype, thereby causing stress tolerance and epigenetic variations that could mask this phenotype [35]. Upregulation of genes related to heat, salt, cold, and UV stress response have been observed in rice due to TE insertions [36]. An insertion of a retrotransposon in soybean led to photoperiod-insensitivity [37]. Thousands of DMRs were identified in this study. Some of the DMRs were found to overlap with TEs. Knowing the biological functions of these DMRs are very important. Only then we can tell, which of these thousands can play important role in developing improved switchgrass. DMRs can potentially affect nearby gene expression and lead to phenotypic variations between AP13 and VS16. Given the benefits of epigenetic variations from some TE polymorphisms, ultimately controlling TE insertion can lead to a valuable plant breeding tool to improve switchgrass and other significant crops.

## Conclusion

Overall DNA methylation levels in the two switchgrass genotypes AP13 and VS16 were found to be similar. CG methylation levels were the highest, followed by CHG, and CHH was the lowest. DNA methylation broadly regulates the genome, as no enrichment of GO terms was found resulting from the BS-seq datasets. The typical pattern of increased DNA methylation was seen in TEs compared to genic regions. MeDIP-seq did not identify differentially methylated regions as effectively as BS-seq, but did effectively locate methylated regions throughout the genome. The A and B subgenomes of switchgrass did not have significantly differential methylation between collinear gene pairs.

## Methods

### Plant material

Switchgrass genotypes, AP13 (lowland ecotype) and VS16 (upland ecotype), were grown in a greenhouse at Delaware State University, Dover, DE with 28 °C day/20 °C night temperature and 12–14 h photoperiod. Plants were grown in 9 in. pots with Pro-Mix Bx soil and watered when soil dried to prevent waterlogging stress.

### DNA extraction

A CTAB-based protocol was used for DNA extraction from leaves of both genotypes (AP13 and VS) [38]. The quality of DNA was determined by running on a 1.0% agarose gel electrophoresis and quantified via a Nanodrop 2000 spectrophotometer (Thermo Scientific, Wilmington, DE). DNA samples were collected separately from the leaves of nine clonal ramets (biological replicates) of each genotype was then pooled in equimolar amounts. A single pooled genomic DNA sample was used for generating MeDIP-seq BS-seq libraries. All libraries were sequenced on the Illumina/HiSeq-2500 platform (Illumina Inc., San Diego, CA).

### Methylated cytosine sequencing

#### BS-sequencing library preparation

Methyl-MaxiSeq™ EpiQuest (BS-seq) libraries were prepared from 100 ng of genomic DNA and underwent bisulfite treatment using Zymo Research EZ DNA Methylation - Lightning™ kit (Cat#: D5030, Zymo Research, Irvine, CA). The resulting bisulfite-converted DNA was PCR- amplified and ligated to adapters, with barcodes. Amplified fragments were purified using the DNA Clean & Concentrator-5™ (Cat#: D4003, Zymo Research, Irvine, CA). The librarieswere checked for size and and concentration using the Agilent 2200 TapeStation instrument (Agilent Technologies, Santa Clara, CA), followed by sequencing on the Illumina HiSeq 2500 platform.

### Processing of BS-Seq reads

Bisulfite-treated libraries were analyzed using the standard Illumina base-calling software. Alignment was conducted with Bismark (http://www.bioinformatics.babraham.ac.uk/projects/bismark/) [39]. Index files were constructed with the *Bismark_genome_preparation* command using the switchgrass reference genome (V2.1). Bismark’s *–non_directional* and other default parameters were applied. Fisher’s exact test for differentially methylated cytosines (DMCs) was performed for each cytosine having a minimum of five aligned sequence reads.

#### MeDIP-sequencing library construction

MeDIP-seq libraries were prepared using the Zymo Research DNA Methylation IP Kit (Cat #D5101, Zymo Research, Irvine, CA). Immunoprecipitated DNA was PCR amplified, purified, and quantified, as described above. Libraries were normalized to 4 nM, followed by sequencing on the Illumina HiSeq 2500 platform (Illumina Inc., San Diego, CA).

#### MeDIP-Seq sequence alignments and data analysis

Reads were aligned to the reference *Panicum virgatum* genome (V2.1), with Bowtie’s best mode and other default parameters. “MACS2 callpeak” was used to call peaks, using input DNA as the control. BIGWIG files were generated from the coverage for visualization purposes [40]. MeDIP-seq peak density was calculated using a previously reported method [41]. All sequences derived from this project were submitted to the short read archives at NCBI, BioProject#PRJNA369416.

### Identification of differentially methylated regions (DMRs) between AP13 and VS16

To study the methylation differences between AP13 and VS16, DMRs were identified. Briefly, Fisher’s exact tests were performed for the cytosines that can be covered by at least 5 reads in AP13 and VS16. Then multiple test correction was performed to control FDR. DMCs were defined as the cytosines with FDR < 0.05. DMCs that had a distance less than 100 bps were merged into clusters. DMCs clusters that had less than 4 DMC and were shorter than 50 bps were discarded. The remaining DMC clusters were then defined as DMRs requiring methylation differences above 0.4, 0.2 and 0.1 for CG, CHG and CHH context, respectively. In other published literature, similar strategies are widely used and no bias was reported [26, 42, 43].

### Distribution of DMRs and peaks across genomic features

DMRs and/or peaks were assigned to genomic features based on gene annotations available from JGI and in-house repeat annotation in GFF3 files. The features include: promoter, CDS, intron, UTR, TEs, 2 kb upstream, and 2 kb downstream of genes.

### Analysis of DNA methylation of two subgenomes in Switchgrass

Proteins from each subgenome were extracted (9 chromosomes of each subgenome A and B). BLASTp was performed using subgenome B as queries against subgenome A [44]. BLAST results and gene annotation information were used as input for MCScanX to identify collinear genes.

#### Repeat annotation

RepeatModeler [45] and RepeatMasker [46] were used to identify repeat families in the switchgrass genome. Repeat families were first identified by RepeatModeler and then used as a library to introduce repeat annotations by RepeatMasker [45, 46].

### Visualization of data

Plots were generated using Circos [47]. Color keys were selected from ColorBrewer (http://colorbrewer2.org) [48]. Figure legends were added into the Circos plots by Inkscape (http://inkscape.org) [49]. ViewBS (http://github.com/readbio/ViewBS) was used to extract information of global DNA methylation levels, distribution of methylation levels, methylation patterns across genes and TEs, DMRs, etc. Figures were also generated via ViewBS. Integrative Genome Viewer (IGV; http://software.broadinstitute.org/software/igv/) was used to visualize MeDIP-seq and BS-seq tracks together [50].

## Additional files

Additional file 1: Figure S1. Meta-plots of DNA methylation level across gene without intronic TE. (PDF 7 kb)

Additional file 2: Table S1. Overrepresentation hypergeometric test on Hypermethylated-DMRs and Hypomethylated-DMRs. (XLSX 9 kb)

Additional file 3: Figure S2. Violin plot of length of TE that were associated with DMRs. (PDF 19 kb)

Additional file 4: Table S2. GO analysis of hypermethylated- and hypomethylated-DMRs. (XLSX 824 kb)

Additional file 5: Table S3. Hypermethylated- and hypomethylated-DMRs flanking within 2000 bp of annotated genes. (XLSX 204 kb)

Additional file 6: Figure S3. Meta-plots of DNA methylation level across collinear gene pairs between two sub genomes in AP13 (A) and VS16 (B). Methylation patterns across genes in AP13 for collinear gene pairs on the two sub genome (A-C). SubA means genes from sub genome A; SubB means genes from sub genome B. (TIFF 137 kb)

Additional file 7: Figure S4. Distribution of methylation levels of common peaks for CG, CHG and CHH context. (PDF 6 kb)

Additional file 8: Figure S5. IGV view of a hypermethylated-DMR (A) and a hypomethylated (B). (TIFF 173 kb)

## Abbreviations

BS-Seq: Whole genome bisulfite-sequencing
CDS: Coding sequence
DMC: Differentially methylated cytosines
DMR: Differentially methylated region
GO: Gene ontology
LINEs: Long interspersed nuclear elements
LTRs: Long terminal repeats
MeDIP-Seq: Methylated immunoprecipitation-sequencing
TE: Transposable element
TSS: Transcription start site
TTS: Transcription termination site
UTR: Untranslated region
