# Supplementary figures and images for: Analyses of methylomes of upland and lowland switchgrass (Panicum virgatum) ecotypes using MeDIP-seq and BS-seq
Source: BMC Genomics. 2017 Nov 7;18:851. doi: 10.1186/s12864-017-4218-0 (PMC5678558; doi:10.1186/s12864-017-4218-0)

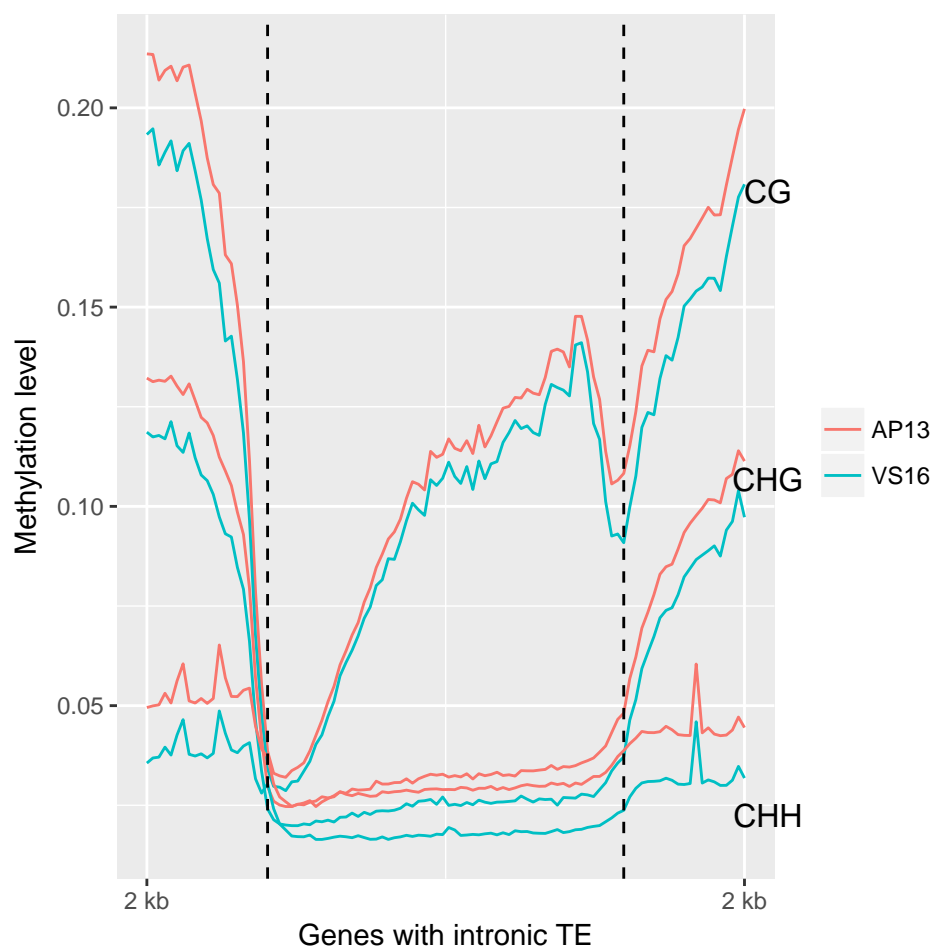

Supplement: Supplementary file 1 — Meta-plots of DNA methylation level across gene without intronic TE. (PDF 7 kb) [file 12864_2017_4218_MOESM1_ESM.pdf]

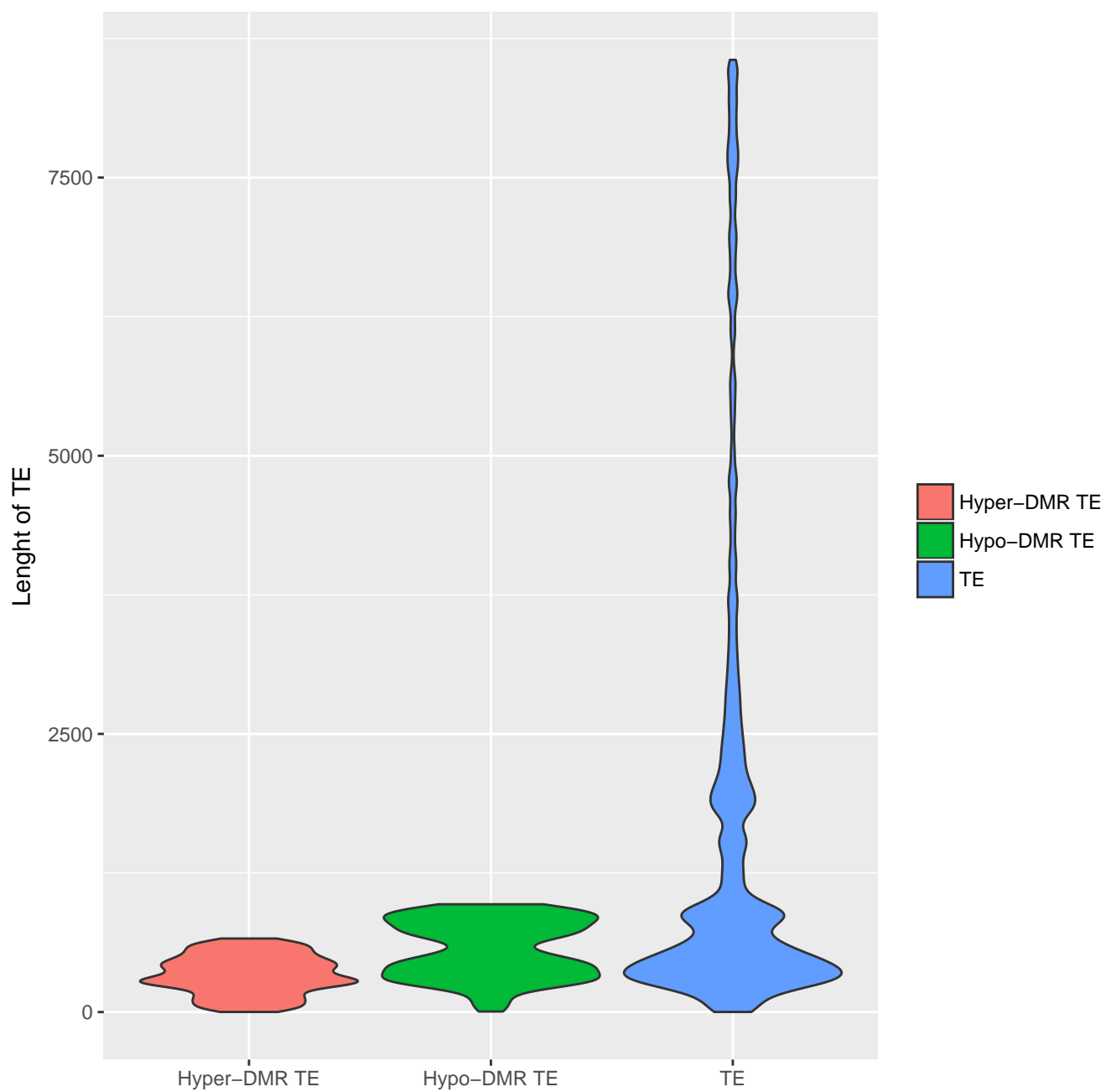

Supplement: Supplementary file 3 — Violin plot of length of TE that were associated with DMRs. (PDF 19 kb) [file 12864_2017_4218_MOESM3_ESM.pdf]

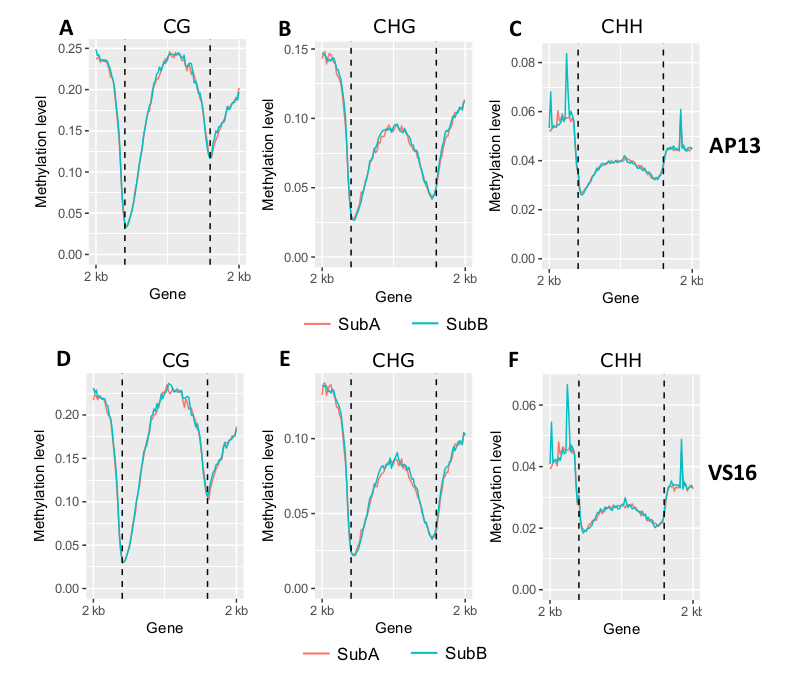

Supplement: Supplementary file 6 — Meta-plots of DNA methylation level across collinear gene pairs between two sub genomes in AP13 (A) and VS16 (B). Methylation patterns across genes in AP13 for collinear gene pairs on the two sub genome (A-C). SubA means genes from sub genome A; SubB means genes from sub genome B. (TIFF 137 kb) [file 12864_2017_4218_MOESM6_ESM.tiff]

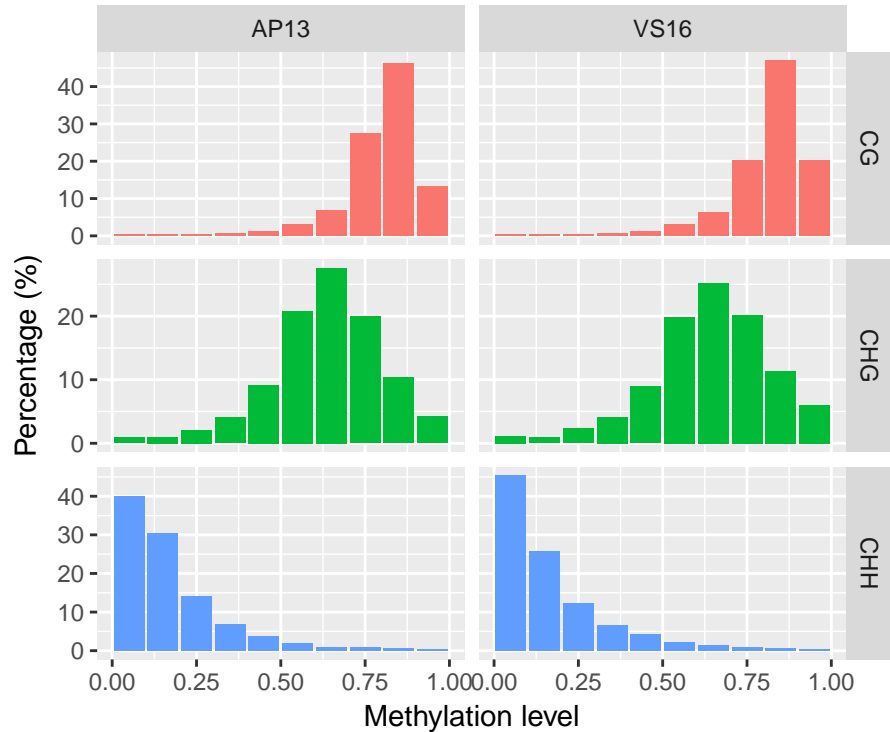

Supplement: Supplementary file 7 — Distribution of methylation levels of common peaks for CG, CHG and CHH context. (PDF 6 kb) [file 12864_2017_4218_MOESM7_ESM.pdf]

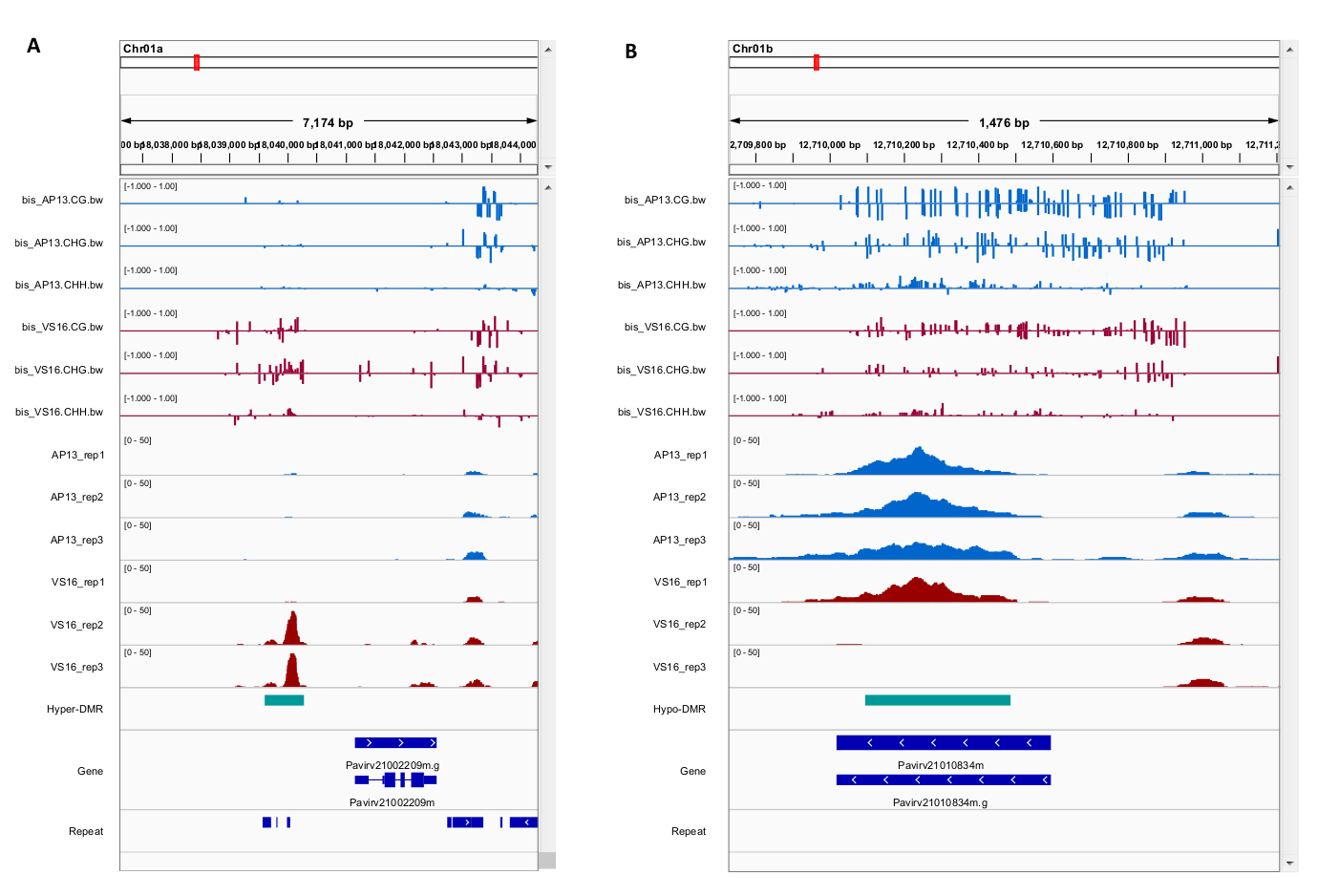

Supplement: Supplementary file 8 — IGV view of a hypermethylated-DMR (A) and a hypomethylated (B). (TIFF 173 kb) [file 12864_2017_4218_MOESM8_ESM.tiff]
